# Advanced Techniques Using In Vivo Electroporation to Study the Molecular Mechanisms of Cerebral Development Disorders

**DOI:** 10.3390/ijms241814128

**Published:** 2023-09-15

**Authors:** Chen Yang, Atsunori Shitamukai, Shucai Yang, Ayano Kawaguchi

**Affiliations:** 1 Human Anatomy and Histology and Embryology, School of Basic Medicine, Harbin Medical University, Harbin 150081, China; 2 Department of Human Morphology, Okayama University Graduate School of Medicine, Density and Pharmaceutical Sciences, Okayama 700-8558, Japan

**Keywords:** in vivo electroporation, in utero electroporation, genome editing, IUE, iON, TEMPO, iGONAD

## Abstract

The mammalian cerebral cortex undergoes a strictly regulated developmental process. Detailed in situ visualizations, imaging of these dynamic processes, and in vivo functional gene studies significantly enhance our understanding of brain development and related disorders. This review introduces basic techniques and recent advancements in in vivo electroporation for investigating the molecular mechanisms underlying cerebral diseases. In utero electroporation (IUE) is extensively used to visualize and modify these processes, including the forced expression of pathological mutants in human diseases; thus, this method can be used to establish animal disease models. The advent of advanced techniques, such as genome editing, including de novo knockout, knock-in, epigenetic editing, and spatiotemporal gene regulation, has further expanded our list of investigative tools. These tools include the iON expression switch for the precise control of timing and copy numbers of exogenous genes and TEMPO for investigating the temporal effects of genes. We also introduce the iGONAD method, an improved genome editing via oviductal nucleic acid delivery approach, as a novel genome-editing technique that has accelerated brain development exploration. These advanced in vivo electroporation methods are expected to provide valuable insights into pathological conditions associated with human brain disorders.

## 1. Introduction

The mammalian cerebral cortex undergoes a tightly regulated developmental process. At the early stage of development, most neural progenitor cells (neural stem cells) undergo proliferative division to expand their population. At the onset of neurogenesis, they start to undergo asymmetric division to produce a neuronally differentiating cell—a neuron or an intermediate progenitor cell (IP). These neuronal progenies initiate basal migration to position themselves within a specific cortical plate (CP) layer, where they adopt a mature morphology to establish proper neuronal circuits. At a later stage, neural progenitor cells gradually shift from neurogenesis to gliogenesis [1,2,3]. The regulatory mechanisms involved in these dynamic processes of neural progenitor cells and their progenies, such as proliferation, migration, differentiation, axon elongation, dendrite development, and the formation of neural circuits, are the fundamental basis for normal brain function [4,5,6]. An accumulating body of evidence demonstrates that pathogenic somatic mutations that occur during development have a role in human cortical malformation during development [7]. Therefore, the detailed visualizations and imaging of these dynamic processes in situ, coupled with functional studies of the related genes in vivo, significantly enhance our understanding of brain development and the pathology of brain developmental disorders.

In utero electroporation (IUE), a kind of in vivo gene delivery method has been widely applied to such visualization and functional studies in developing and postnatal brains [8,9,10]. The recent advent of advanced techniques such as spatiotemporal gene regulation [11] and genome editing [12,13] have been applied to IUE and further expanded our investigative toolbox. In addition to IUE, the iGONAD method, a newly developed genome-editing approach by intragonadal electroporation, is an accelerated method to explore brain development [14,15]. It has become possible to create genome-edited animals without the need for the in vitro manipulation of fertilized eggs.

In this review, we present an overview of the numerous in vivo electroporation techniques, especially recent advanced techniques that can be applied to investigate the molecular mechanisms underlying cerebral disorders and diseases.

## 2. Basic Technique for IUE

**Figure 1:**
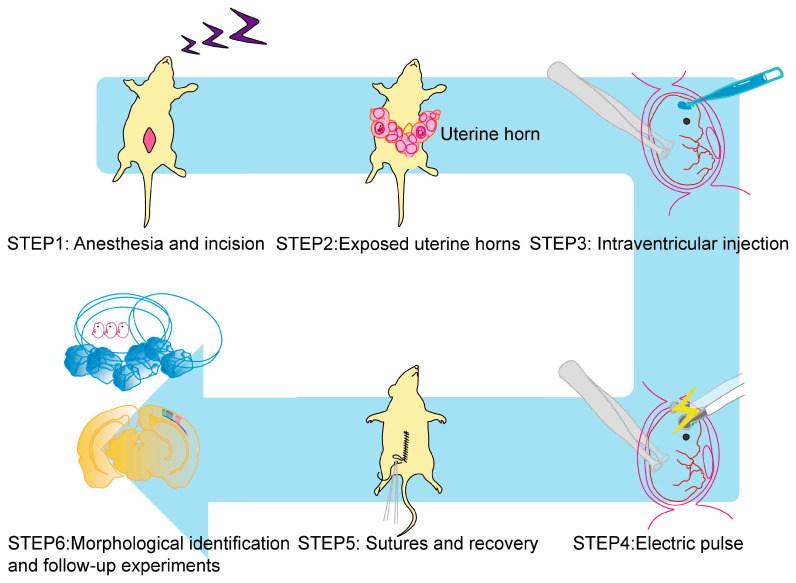
Basic steps for IUE to mouse embryos.

In the basic IUE strategy (Figure 1), a pregnant mouse is anesthetized, and the plasmid DNA vector solution is injected into the embryo’s lateral ventricle using a glass microcapillary through the uterine wall. Then, the forceps-type electrodes apply the electrical pulses to derive DNAs into the inside of the cells aligned to the ventricular surface [8,9]. Post-electroporation, the uterine horns are reinserted for normal embryonic development. The introduction of plasmid vectors usually targets the dorsolateral cerebral area, although other cortical areas can also be targeted by adjusting the electrode angle [16,17].

IUE exhibits a preference for the induction of exogenous genes into cells whose cell bodies lie on the surface of the ventricles. These cells are neural progenitor cells and daughter cells that are newly born at the time of electroporation. This property implies that when the different plasmid vectors are mixed and introduced, they are introduced into almost the same cells [8,9]. As such, the co-electroporation of an EGFP expression vector is commonly used to label cells that have undergone electroporation.

### 2.1. Developmental Stage and Animals

IUE protocols have been optimized for each developmental stage, including the size of the electrode and the applied voltage. For mouse embryos, IUE has been reported to be applicable from embryonic day (E) 12 to E17 [9] and from E11 to E15 [8]. IUE to E9 and E10 mouse embryos can be successfully performed by introducing an improved light source that enhances the visibility of embryos through the uterine wall [18]. While electroporation parameters may vary across different research laboratories, we performed under the condition of: voltage: E10–11, 50 V; E12–E14, 32–35 V, 50 msec ON, 450 msec OFF, 4–5 pulses, with electrode size: E10–11, 1 mm; E12–E14, 3 mm [19,20]. IUE was also used to introduce the plasmid vectors at various times (for example, two times at different embryonic stages of the same embryo) [21]. After birth, postnatal brain analysis is also feasible [9].

In addition to mice and rats, electroporation techniques can also be applied to the embryonic brains of other animals, including in ovo applications to birds [22,23] and reptiles [24,25], in pouch applications to dunnarts [26], in utero applications to guinea pigs [27] and ferrets [28,29].

### 2.2. Selection of Promoters for Exogenous Gene Expression

#### 2.2.1. Ubiquitous Promoters

IUE is frequently used to visualize the cells in the developing brain or induce gene expression to study its function. Ubiquitous promoters, such as the CAG promoter, the human elongation Factor 1 α (EF1α) promoter, and the cytomegalovirus (CMV) promoter [9], are commonly employed for these purposes. It is worth noting subtle differences, however, as the CAG promoter shows a slightly higher level of functionality in neurons than in neural progenitor cells when compared to EF1α [20]. Additionally, the CMV promoter is silenced in mature neurons in postnatal brains [9].

#### 2.2.2. Specific Promoters

Cell type-specific promoters are also used in IUE. For target neural progenitor cells, the Nestin promoter or enhancer [30], the BLBP promoter [31,32], and the human glial fibrillary acidic protein (GFAP) promoter have been used, while the mouse GFAP promoter is often utilized for labeling astrocytes [33].

To start gene expression in the earliest neuronally differentiating cells in the embryonic cerebral walls, researchers can use a Gadd45-gamma promoter [34]. The Tubulin alpha 1 promoter has been used to target early neuronally differentiating cells, including IPs [35]. The NeuroD promoter starts to work slightly later [36]. The Tbr1 promoter [37] starts in more differentiated neurons than the NeuroD promoter [36].

In all cases, cell type-specific promoters have the problem of leaky expression [38], whereby the promoter drives gene expression in unintended cell types. Therefore, methods have been developed for more stringent control of expression in specific cell types and timing, which will be described in the latter part of this review.

### 2.3. Pathological Mutant Analysis by IUE

Dynamic processes in neural progenitor cells that are crucial for brain development are regulated by complex mechanisms. Disruptions can lead to disorders classified as malformations of cortical development (MCD). In studying human MCDs, IUE experiments have proven particularly compatible with research into diseases caused by dominant negative and gain-of-function mutations. One such disorder classified under MCD is periventricular nodular heterotopia (PNH). PNH, a familial disorder, has been linked to mutations in the *NEDD4L* gene, and forced expression of mutant NEDD4L by IUE induces increased proliferation of neural progenitor cells and impaired neuronal migration and positioning, indicating the significant impact of *NEDD4L* mutation on neurodevelopmental processes [39].

Some somatic mutations occurring during development also have a pathogenic effect and can contribute to some epileptic malformations of cortical development and autism spectrum disorder [7]. Thus, the misexpression of such mutant proteins via IUE can be utilized to generate an animal model for brain diseases. Postzygotic somatic mutations that activate the PI3K-AKT-mTOR pathways are found in a wide range of brain diseases, including focal malformations of cortical development (FMCDs), which account for the majority of drug-resistant pediatric epilepsy cases. Introducing the AKT3E17K FMCD-associated mutant into mouse brains by IUE resulted in electrographic seizures and impaired hemispheric architecture, thereby providing an animal model for FMCDs [40].

Another example is related to de novo tumorigenesis. The exogenous expression of the human truncated PPM1d, which is found in pediatric high-grade gliomas, by IUE is sufficient to promote glioma formation in the mouse brain [41].

### 2.4. Loss-of-Function Studies to Investigate the Pathology of Brain Development Disorders

Loss-of-function studies by RNA interference (RNAi) using IUE have also been used to elucidate the molecular mechanisms of certain brain pathologies. For example, knockdown (KD) of *disrupted in schizophrenia 1* (*DISC1*), a gene associated with a variety of psychiatric conditions, including schizophrenia, bipolar disorder, major depression, and autism [42,43], by RNAi via IUE, impairs neural progenitor proliferation [44], neuronal migration and integration [45,46].

Technically, RNAi takes more than a day to downregulate the expression level of the target protein after IUE, although this can vary depending on the type of protein. Regarding this point, the direct introduction of double-stranded short interfering RNA (siRNA) exerts its effect earlier than plasmid vectors expressing short hairpin RNA (shRNA) under a Pol III-type promoter, such as the human U6 promoter [47] in IUE.

### 2.5. Application of Electroporation to Organoid Disease Model

Recently developed research methods using human cell-derived organoids could significantly mitigate the reliance on animal models and serve as accurate models for human conditions. Electroporation is widely used as a convenient method for introducing DNA to the organoids.

Brain organoids, derived from human embryonic stem cells (hESCs) or induced pluripotent stem cells (iPSCs), have emerged as a groundbreaking tool for investigating the early stages of human brain development [48,49]. Brain organoids offer significant advantages as disease models, particularly when derived from patient-specific iPSCs, enabling a more accurate representation of pathological conditions. Electroporation applies to the brain or cerebral organoids, wherein a plasmid solution is injected into the organoid, and electroporation is performed in a cuvette or chamber [50,51]. This method has been employed in creating glioblastoma models [52], miRNA delivery to study heterotopia [53], and the visualization of dendritic architecture and axon formation for live imaging analyses [54]. A similar technique has been applied to the human retina organoid for live-cell imaging [55], expanding its applicability beyond the central nervous system.

### 2.6. Limitations of IUE

IUE comes with certain limitations. Primarily, controlling the expression levels of exogenous genes in individual cells using IUE poses significant challenges [56]. This makes it difficult to perform simple quantitative analysis using reporter gene expression levels. Therefore, when using Ca^2+^ indicators such as GCaMP [57,58] or measuring FRET as reporters expressed by IUE [59], it is necessary to observe changes in the individual cells and evaluate them in terms of ratios or similar quantitative measures. There have been efforts to overcome this disadvantage of IUE using the advanced technique, iON [60], as described in the latter part of this review. Manipulating specific cells, such as microglia and vascular endothelial cells, which are crucial in studying neurological disorders, also presents difficulty. Finally, introducing exogenous plasmid DNA could elicit a microglial response via Toll-like receptor 9 (TLR9) activation [61], a factor that warrants careful consideration when interpreting microglial behavior post-IUE. However, co-injection of a TLR9 antagonist, oligonucleotide 2088, with plasmid DNA vectors can mitigate this response [61].

## 3. Genome Editing by IUE

### 3.1. Knockout (KO)

**Figure 2:**
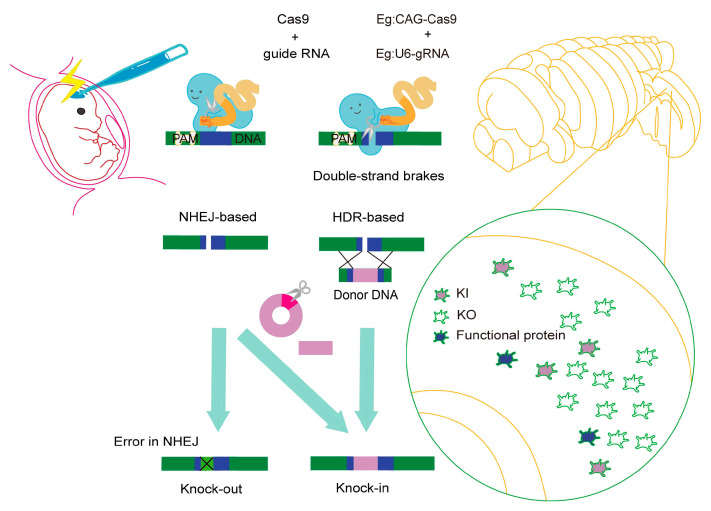
In vivo genome editing with Cas9-initiated double-strand brakes (DSBs) via IUE. A key consideration for in vivo genome editing via IUE is that not all cells undergo a KI event. In those cells that do not, gene locus mutations or deletions are commonly observed.

To bypass the off-target effects of RNAi and ensure the complete removal of the target protein in neural progenitor cells and their descendants, a knockout (KO) strategy is pivotal. Genome editing via IUE allows for cell-level knockout in developing brains (Figure 2). This is effectively achieved through the induction of insertions or deletions (indels) mediated by the nonhomologous end joining (NHEJ) pathway of CRISPR/Cas9 during IUE [62,63,64]. In the experimental steps, typically, plasmid vectors expressing the Cas9 protein and guide RNA(s) are coexpressed by IUE. When genome editing is successfully performed in neural progenitor cells, their progeny also become knockouts (Figure 2). Thus, the piggyBac system is occasionally utilized to visualize electroporated cells and their progeny; in this system, a ubiquitous promoter with marker gene cDNAs, such as EGFP, is integrated into the genome [65].

Similar to in vitro cultured cell genome editing, a crucial point to note is the need to validate whether the target protein has been effectively knocked out. This validation of gene perturbation ultimately relies on immunohistochemistry-based analysis using antibodies [66]. However, in the case of IUE, while evaluating the overall efficiency of knockout is possible, it becomes particularly challenging to definitively assess the knockout status on a cell-by-cell basis. This difficulty arises from the inherent cell-to-cell variation in the efficiency of genome editing with IUE and potential instances of partial protein deletion. A knock-in method is also being used to improve KO efficiency and accuracy, which involves the insertion of stop codon repeat sequences by homology-directed repair (HDR) [67]. To better understand the knockout status in each cell, high-throughput genotyping at the single-cell level is beneficial [68]. However, this approach may only sometimes be feasible in practice.

Nevertheless, due to its relative simplicity, genome editing by IUE is a feasible strategy not only in common laboratory animals such as mice and rats but also in other species. As long as a degree of genome information is known, this procedure can be implemented in various species, such as ferrets [19,69].

### 3.2. Knock-In (KI)

Knock-ins (KI) initiated by Cas9’s double-strand break (DSB) are also conducted using IUE. While the efficiency is high enough for inserting small tags, there is still room for improvement regarding the efficiency of long DNA insertions, such as cDNAs for fluorescent proteins, Cre recombinase, and various other proteins.

Cell cycle-dependent homology-directed repair (HDR) is commonly performed for KI in neural progenitor cells in vivo via IUE [70,71,72]. This includes various modified methods such as microhomology-mediated end joining (MMEJ), which uses a donor with a sgRNA target site and microhomology arms (5–25 bp), and homology-mediated end joining (HMEJ), which uses a donor with sgRNA target sites plus long homology arms (800 bp) [73]. NHEJ-based knock-in has also been applied in IUE [74], in which nucleotides without homology arms are used as donors (Figure 2).

Several strategies with the donor DNA serving as the template for KI are being explored. Single-stranded oligodeoxynucleotides (ssODNs) [70], long single-stranded DNA [75,76], plasmid DNA [71,72], and adeno-associated virus (AAV) vectors [77] are being utilized. Many efforts are being made to enhance recombination efficiency, such as by introducing sgRNA target sites to cleave both ends of the donor genes in vivo [78]. It should be noted that unexpected leaky expression from the donor plasmid has been reported to occur following IUE, leading the authors to develop a leakless targeting vector to address this issue [71]

Yang et al. conducted comparative analyses of KI efficiencies in IUE mouse embryos using homologous recombination (HR), MMEJ, HMEJ, and NHEJ. These studies have provided valuable insights [73,79]. As a cost-effective strategy, they proposed using linear double-stranded DNA as a donor in Tild-CRISPR [79]. Utilizing this method, Tabata et al. reported the KI of Cre recombinase at the *Olig2* gene locus via IUE [80].

Regarding KIs initiated by Cas9 DSBs, it is important to note that KI does not occur in all cells. In many cells where KI has not occurred, mutations or deletions at the gene locus are frequently observed (Figure 2). This is a prevalent problem in KI using Cas9. To address this, active research is being conducted on inserting DNA at specific sites without DSBs. This includes DNA sequence insertion using the Prime editor, a type of DNA base editor [81], the insertion of transposase recognition sequences by the Prime editor and subsequent transposase recruitment [82], and the use of crRNA-dependent transposase, which is involved in CRISPR array formation [83,84], for targeted large DNA insertion into the genome. While there are challenges regarding KI efficiency, the complexity of the introduced components, and the strict control of insertion sites, the application of these techniques in IUE is expected in the future.

### 3.3. In Vivo Epigenetic Editing in a Brain Disease Model

In vivo epigenome editing via IUE has been conducted to study the role of specific genes and epigenetic marks in brain development and disease. A nuclease-deficient form of Cas9 (dCas9), bound to a transcriptional activator or repressor, has been used for this purpose. For instance, Albert et al. expressed dCas9 fused to the H3K27me3 histone methyltransferase Ezh2, along with a guide RNA (gRNA) targeting one of the CpG islands near the transcription start site of Eomes (Tbr2), a transcription factor involved in neural development. They introduced this system into neural progenitor cells via IUE and successfully observed a reduction in the proportion of Eomes-positive cells. This suggests that their system induces gene repression through the deposition of repressive H3K27me3 marks [85].

To enhance the effectiveness of epigenetic modification, Peter et al. utilized the SunTag system [86]. The authors found that C11orf46 haploinsufficiency was associated with hypoplasia of the corpus callosum in humans [87]. Using a dCas9-SunTag system with C11orf46 binding, they performed epigenome editing of the neurite-regulating genes Sema6a-A/D, which successfully rescued transcallosal dysconnectivity in a mouse model [87]. This provides evidence for the therapeutic potential of in vivo epigenome editing.

## 4. Spatiotemporal Expression Control and Lineage Tracing by IUE

The need to control spatiotemporal expression utilizing IUE is common across diverse areas of study. The conventionally employed expression induction methods or conditional gene expression systems, such as Tet-on, Cre/lox, and CreER combined with tamoxifen, generally apply in IUE.

Long-term labeling transposon-based methods are often utilized for the lineage tracing of neural progenitor cells or for examining the long-term effects of gene manipulation. Transposons, such as piggyBac [88,89], Sleeping Beauty (SB) [90,91], and Tol2 [92], can be implemented to integrate an exogenous gene into the genome of the host, providing stable, long-term gene expression. The combination of piggyBac/Tol2 transposition and Cre/lox recombination expressed by IUE could be utilized for multicolor fate mapping in the developing brain [93,94]. SB with IUE has been used to induce glioma in the postnatal mouse brain [95], providing a tool for functional analysis of the candidate genes in glioma.

### 4.1. Sparse Labeling and Live Imaging

A significantly diluted Cre expression vector plasmid combined with the Cre/lox system allows for nearly clonal labeling by IUE [96]. We have employed this sparse labeling technique with brain slice methodologies [97,98,99] to conduct live imaging analyses of neural stem cell behavior [19,100,101]. Live imaging of the neural cells in brain slices after IUE has also been utilized in various other contexts, such as the analysis of neuronal migration [9,102], and extended to the subcellular level, such as the study of microtubule dynamics using EB3 [103], the Golgi apparatus [104], and visualization of the apical junctional components and centrosomes [59]. These advanced imaging techniques provide invaluable insights into human genetic disorders like lissencephaly, furthering our understanding of their underlying mechanisms.

### 4.2. iON Expression Switch

**Figure 3:**
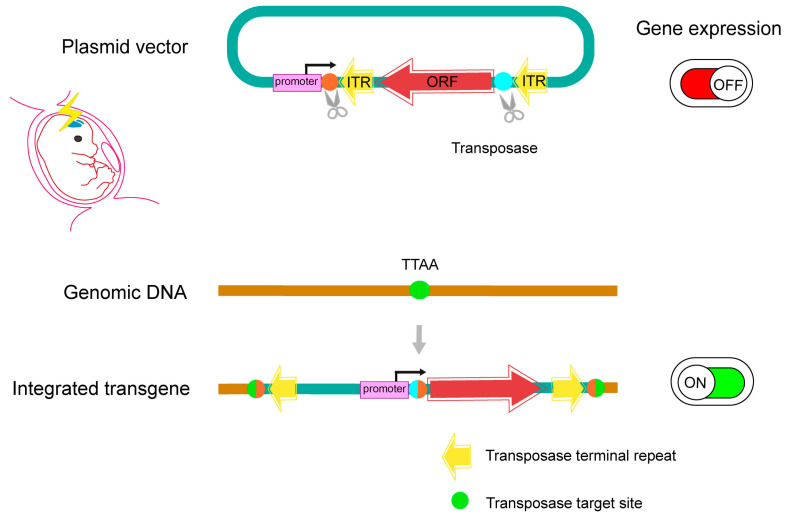
Schematic representation of the iON switch. The iON vector is designed such that the gene of interest is not expressed before it is repaired and integrated into the genome by the transposase. This system is applicable in vivo by IUE in addition to in vitro. ITR, inverted terminal repeat.

Recently, techniques have been developed to control the timing and copy numbers of exogenous genes more precisely. In IUE with transposon systems such as piggyBac, the expression from episomal plasmids immediately after introduction was difficult to control, and there were problems such as the need to wait for dilution to an appropriate level through cell division and the intense maintenance of expression levels in nondividing differentiated cells. These problems were addressed by integration-coupled ON (iON) switch technology, which initiates gene expression upon integration into the genome (Figure 3) [17,60].

iON utilizes the principle of piggyBac transposase-mediated cleavage of inverted terminal repeat (ITR) sequences and directional integration, as well as the DNA repair of cleavage sites in vivo. After cutting the connection between the promoter and the inverted ORF, repair occurred during directional integration into the genome, resulting in the inversion and reconstitution of the promoter and ORF, thereby activating expression. Therefore, iON plasmids with inverted ORFs are not expressed until reconstitution occurs (Figure 3). Some minimal expression from the upstream ITR of the inverted ORF can be observed in iON; thus, a refinement over this was achieved with LiON, where the cleavage site of the connection was located immediately after the ATG start codon. This adjustment allows the remaining ORF portion to be inverted and expressed, reducing the amount of expression from episomal plasmids to near zero.

These systems overcome the disadvantage of IUE, which involves transient overexpression. They allow for stable copy numbers integrated into the genome to be inherited by daughter cells. These improvements prevent toxicity caused by transient overexpression and abnormal protein localization due to excessive expression. Moreover, they address issues such as insufficient expression due to low copy numbers in genome knock-in mice lines.

Utilizing these properties, stable and random combinations of multiple fluorescent proteins have been achieved, following the same principle as Brainbow [105,106]. This approach has improved clonal resolution and has applications in multicolor lineage labeling.

### 4.3. TEMPO

In brain development, the types of differentiated cells produced from neural stem cells change over time. Abnormalities arising in neural stem cells can be apparent in a cell-type-specific manner, potentially leading to the onset of various diseases. Therefore, robust methodologies that enable the investigation of temporal effects are essential. As the typical method is to use a CreER mouse line activated by tamoxifen, a valuable approach exists for modulating and manipulating the expression of target genes within specific time windows. While sequential IUE methods have been reported [20,21], they are often associated with technical difficulty and the potential for cumulative surgical interventions.

**Figure 4:**
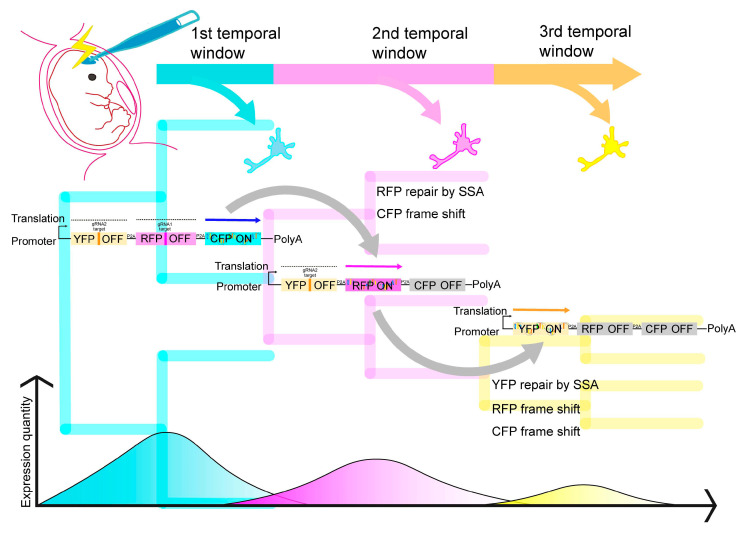
Schematic representation of TEMPO. The TEMPO system for sequential labeling via IUE enables single-cell lineage analysis in the developing cerebrum. ORF, open reading frame; SSA, single-strand annealing.

To overcome these challenges, an innovative technique called TEMPO has been developed [11] (Figure 4). TEMPO harnesses the CRISPR/Cas9 system to induce DSBs, which are then repaired using the highly accurate single-strand annealing (SSA) machinery, an endogenous DNA repair mechanism.

The TEMPO system is composed of gRNA cascades, and the expression cassette consists of ORFs linked in tandem using a 2A peptide (such as the TEMPO reporter cascade shown in Figure 4). Initially, the gRNA cascades activate only the first gRNA, while the others are made inactive through the insertion of a modified RNA pol III transcription termination sequence. The first gRNA specifically targets the insertion site of the second gRNA and becomes active upon the removal of this insertion during SSA repair. The expression cassette includes tandemly linked ORFs using a 2A peptide. Initially, the forward ORF is inactive due to frame-shift mutations, with only the final ORF being expressed (CFP in Figure 4). However, the first gRNA targets the forward ORF and is precisely repaired through SSA, restoring the correct reading frame and disrupting the downstream ORF due to the frame-shift mutation. As a result, a switch in expression occurs from the downstream ORF to the forward ORF (RFP to YFP in Figure 4).

This innovative system enables the sequential manipulation of molecular factors in the developing brain. In mice, successful sequential expression of up to four distinct genes has been achieved with intervals of approximately 24–48 h. The cutting and repair cycles are influenced by the frequency of cell division, with a reduction in frequency leading to a decrease in the efficiency of expression switching.

## 5. iGONAD

**Figure 5:**
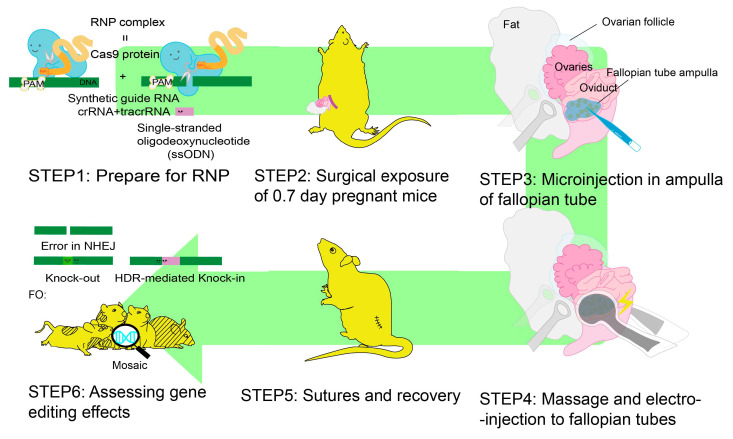
Experimental steps of iGONAD to generate KO or KI mice line. The CRISPR reagent, which includes the ribonucleoprotein (RNP) complex (Cas9 protein and guide RNA) with a repair DNA template (in case of KI), is injected into the ampulla of pregnant day 0.7 female mice. Electroporation was performed to deliver the components into the zygotes.

A technique similar to IUE can be performed for genome editing of the early stage embryos in the oviduct (Figure 5). This technique allows rapid, easy preparation to make the KO animals by NHEJ-mediated indels events and the KI animals by HDR.

Traditional gene-targeted animal generation protocols involve the following three standard steps: the isolation of embryos (fertilized eggs/blastocysts) from donor females; the injection of genetic modification reagents (DNA or ES cells) into the embryos by microinjection followed by brief culture in vitro; and the transfer of treated embryos into recipient females. iGONAD, improved Genome editing via Oviductal Nucleic Acids Delivery [15,107], which improved the original GONAD method [108], does not require any of the three main steps.

Figure 5 summarizes the experimental steps involved in iGONAD [109]. The CRISPR reagent, which includes the RNP complex (complex of Cas9 protein and guide RNA) with a donor DNA template (in the case of KI), is injected into the ampulla of pregnant day 0.7 female mide when fertilized eggs exit the oocyte envelope in the oviductal ampulla. Then, electroporation is performed to deliver the components into the zygotes (Figure 5). Table 1 lists parameters for electroporation under the various experimental conditions introduced in this review. These procedures can be performed in laboratories lacking sophisticated microinjection equipment by researchers skilled in small animal surgery. Furthermore, iGONAD does not require vas deferens removal in males and pseudopregnant females. This allows female animals to be used multiple times for further GONAD procedures [109], accelerating the development of genetically engineered animal models. Beyond just mice and rats, the applicability of these methods extends to other animal models, enhancing their potential impact by contributing to a more diverse range of genetically engineered animal lines for research.

Similar to genome editing by IUE, iGONAD also allows for the creation of both KO embryos through NHEJ-mediated indel events and KI embryos through HDR-mediated events. Researchers have successfully produced HDR-mediated KI animals via iGONAD with long ssDNA (−1 kb) and ssODNs as repair template DNA [110]. The efficiency of generating KI animals by genome editing has improved with the use of ssODN as the donor nucleic acid [75,111]; however, electroporation remains less efficient than microinjection [112]. This limitation of iGONAD, especially when aiming for long-sequence knock-ins, suggests that further refinement is needed in future research.

In practice, precise control over the timing of mating is crucial for the success of iGONAD. However, this can pose some difficulties at the laboratory level, as mating must be scheduled during nighttime hours, which often leads to discrepancies of several hours. Since mosaicism and variation among embryos often occur, when mosaicism is unsuitable for analysis, these animals need to be bred with wild-type individuals to establish a lineage for subsequent analyses. Additionally, we are conducting experiments with IUE in F0 embryos generated by iGONAD; however, we cannot ascertain which embryos have been genome-edited at the time of IUE. The success of the experimental procedure depends not only on the timing of mating but also on the target genome sequence. Consequently, multiple conditions may need to be tested for the best outcomes.

## 6. Perspectives

These advanced methods of in vivo electroporation are expected to provide valuable information that will yield crucial insights that could inform the understanding of pathological conditions linked to human brain disorders. In addition to IUE in embryonic brains, the refinement of in vivo electroporation techniques for postnatal and adult brains is underway. Using a whole-brain scale with plate electrodes placed around the animal’s head or microelectrodes inserted into the brain [113,114,115], exogenous gene expression can be induced in the neurons, neural stem cells (NSCs), or astrocytes in postnatal and adult brains. Advanced techniques such as genome editing and spatiotemporal control, as described in this review, are also basically applicable to these stages.

Coinciding with advances in in vivo electroporation, the development of superresolution microscopy and expansion microscopy techniques has enabled the observation of molecular localization at the nanoscale level [116,117,118,119]. This has enhanced the effectiveness of molecular tagging by KI. Thus, particularly in brain development research, where there is sometimes a desire to analyze the localization of molecules at the nanoscale within a tissue, the KI technique by in vivo electroporation has become an invaluable toolset for accelerating research. 

The techniques of iGONAD and IUE bear a close resemblance to each other in terms of experimental procedures, and the equipment used is largely the same. However, generating transgenic animals using methods like those involving piggyBac systems in IUE still remains a significant challenge in iGONAD. The prospect of creating transgenic animals using methods similar to iGONAD, therefore, represents a significant potential advancement for research tools in the study of neuronal development disorders.

Regarding the lineage tracing of neural progenitors in vivo, a genetic labeling method has been developed that involves an intraventricular injection of a retroviral library containing genetic barcodes. This approach enables the unique labeling of each transduced cell, facilitating the study of clonal dynamics [120], such as in glioma progression [121]. With future technological advancements, IUE may be adopted for genetic barcode labeling and other recently developed molecular recorders to track various cellular events [122,123,124,125,126].

## Figures and Tables

**Table 1:** Parameters for electroporation.

Animal Species	Stage, Sample	Voltage (V)	On Time (msec)	Off Time (msec)	Pulse Number	Electrode Size (mm)	References
Mouse	E(embryonic day) 9–E17	25–50	50	450–950	4–5	1–5	[8,9,18,19,20,21]
Rat	E13–14	65	1	–	–	10 x 5	[58]
Chick	HH10 (1.5 dpo [day post-ovoposition])	25	50	950	5	0.5 x 1.0	[22]
Chick	4 dpo	30	5	500	5	3	[23]
Snake	4 dpo	30	5	500	5	3	[25]
Turtle	14 dpo	32	50	950	2	needle type (CUY200S, NEPAGENE, Japan)	[24]
Gecko	14 dpo	32	50	950	2	needle type (CUY200S, NEPAGENE, Japan)	[24]
Dunnart	Stage20 (Postnatal day 8–11)	30–35	100	900	5	1	[26]
Guinea pig	E28–37	40–54	50	950	4	5	[27]
Ferret	E32	45	100	900	5	10	[19]
Ferret	E35–E38	50–100	50	950	5	5	[28,29]
Human brain organoid	20–40 days in culture	80	50	500–950	5	chamber	[51,53,54]
Human brain organoid	20–40 days in culture	–	–	–	–	cuvette (Nucleofector, A-23 program, LONZA, USA)	[50]
Human brain organoid	4 months in culture	45	50	950	5	3	[52]
Human retinal organoid	27 days in culture	25	50	950	5	chamber	[55]
Mouse (GONAD/iGONAD) step1	E0.7–1.5	50, 10% decay	5	50	3	3	[14,15,108,109]
Mouse (GONAD/iGONAD) step2	E0.7–1.5	10, 40% decay	50	50	3	3	[14,15,108,109]

## Data Availability

Not applicable.
